# Effects of Astaxanthin supplementation on selected metabolic parameters, anthropometric indices, Sirtuin1 and TNF-α levels in patients with coronary artery disease: A randomized, double-blind, placebo-controlled clinical trial

**DOI:** 10.3389/fnut.2023.1104169

**Published:** 2023-03-27

**Authors:** Marzieh Heidari, Maryam Chaboksafar, Mohammad Alizadeh, Bahram Sohrabi, Sorayya Kheirouri

**Affiliations:** ^1^Department of Clinical Nutrition, Faculty of Nutrition and Food Science, Tabriz University of Medical Sciences, Tabriz, Iran; ^2^Cardiovascular Research Center, Tabriz University of Medical Sciences, Tabriz, Iran; ^3^Department of Nutrition, Faculty of Nutrition, Tabriz University of Medical Sciences, Tabriz, Iran

**Keywords:** coronary artery disease, astaxanthin, lipid profile, glycemic indices, inflammation

## Abstract

**Background:**

Atherosclerosis can develop as a result of an increase in oxidative stress and concurrently rising levels of inflammation. Astaxanthin (AX), a red fat-soluble pigment classified as a xanthophyll, may be able to prevent the vascular damage induced by free radicals and the activation of inflammatory signaling pathways. The objective of the current study is to assess the effects of AX supplementation on cardiometabolic risk factors in individuals with coronary artery disease (CAD).

**Methods:**

This randomized double-blind placebo-controlled clinical trial was conducted among 50 CAD patients. Participants were randomly allocated into two groups to intake either AX supplements (12 mg/day) or placebo for 8 weeks. Lipid profile, glycemic parameters, anthropometric indices, body composition, Siruin1 and TNF-α levels were measured at baseline and after 8 weeks.

**Results:**

Body composition, glycemic indices, serum levels of TNF-α, Sirtuin1 did not differ substantially between the AX and placebo groups (*p* > 0.05). The data of AX group showed significant reduction in total cholesterol (−14.95 ± 33.57 mg/dl, *p* < 0.05) and LDL-C (−14.64 ± 28.27 mg/dl, p < 0.05). However, TG and HDL-C levels could not be affected through AX supplementation.

**Conclusion:**

Our results suggest that AX supplementation play a beneficial role in reducing some components of lipid profile levels. However, further clinical investigations in CAD patients are required to obtain more conclusive findings.

**Clinical trial registration:**

www.Irct.ir., identifier IRCT20201227049857N1.

## Introduction

Coronary artery disease (CAD) is the leading cause of morbidity, disability and death worldwide (1). Atherosclerosis as the primary cause of CAD is initiated with the damage and subsequent impairment of the functional integrity of the vascular endothelium (2). Endothelial dysfunction is a result of oxidative stress which occurs concurrently with the activation of pro-inflammatory signaling pathways and the release of cytokines and chemokines (3, 4). The modifiable risk factors for CAD include hypertension, insulin resistance, dyslipidemia, chronic inflammation, obesity and sedentary lifestyle (5, 6). The high cost and side effects of synthetic drugs have drawn attention to the use of dietary supplements and antioxidants, that might be an easy way to restore the redox state and inhibiting the oxidative stress (7, 8).

Astaxanthin (AX) is a fat-soluble carotenoid without provitamin A activity in humans (9). It belongs to xanthophylls with the chemical formula C40H52O4 (10). The natural sources of AX found in various microorganisms and marine animals including microalgae, yeast, salmon, trout, shrimp, lobster, and crayfish (11, 12). One of the most important sources of biological AX is green unicellular microalga H.pluvialis which contains more than 80% AX content in its cells (13, 14). Affirmative impacts of AX are often associated with its anti-inflammatory, antioxidative, and antiapoptotic properties (15). Some of clinical trial studies have found that AX can improve metabolic markers such as lipid profile or glycemic indices (16–18). Urakaze et al. investigated that the supplementation of 12 mg AX for 12 weeks in subjects with prediabetes lead to significant reduction in malondialdehyde-modified low-density lipoprotein, glucose levels after 120 min in oral glucose tolerance test and HbA1c (Hemoglobin A1C) compared with placebo group (19). According to an vivo study, AX consumption in ApoE^−^/^−^ mice model caused a significant decline in total cholesterol (TC) and triglycerides (TG), as well as aortic atherosclerotic plaque levels and aortic cholesterol levels (20). AX has attracted increasing interest as a multi-target pharmacological agent and it has been suggested that AX may be able to be a potential therapeutic agent against atherosclerotic cardiovascular disease (21, 22). However, according to our knowledge, no study has been conducted on the effect of AX supplementation in CAD patients. Therefore, the purpose of this study was to evaluate the effect of AX supplementation on body composition, metabolic and anthropometric parameters in patients with CAD.

## Materials and methods

### Study design and participants

This parallel-group, randomized double-blind, placebo-controlled clinical trial, registered in Iranian Registry of Clinical Trials (http://www.irct.ir: IRCT20201227049857N1). CONSORT 2010 guideline was implemented in the design of the study. CAD patients were selected between December 2020 and June 2021. The Tabriz University of Medical Sciences Research Ethics Committee accepted the study protocol, and this study was conducted in compliance with the Declaration of Helsinki.

Inclusion criteria included men or women 40–65 years old, angiographic evidence of 50% stenosis in at least one of the major coronary arteries, 25 < BMI (in kg/m^2^) <35, ability and willingness to cooperate in the trial. Exclusion criteria included consumption of alcohol, hookah and smoking; hypothyroidism and hyperthyroidism; uncontrolled diabetes; history of myocardial infarction; disorders in heart function (classes of 3 and 4), valvular heart disease, renal failure, breastfeeding or pregnancy, consumption of herbal and antioxidant supplements in the last 2 months.

In total, 162 patients were screened for inclusion and 50 participants were randomly allocated into either 12 mg AX or placebo (microcrystalline cellulose) groups along with low calorie diet, for a period of 8 weeks. Additionally, nutritional recommendations were offered along with the low-calorie diet, which was designed based on the patient’s preferences and calorie requirements. Phone calls were made once a week to check on the participants and make sure the intervention procedure was being followed. In addition, the individuals had appointments every 2 weeks during the supplementation period. Consuming ≥90% of supplements was considered treatment compliance.

### Randomization and blinding

Eligible participants were randomly allocated to treatment groups in a 1:1 ratio using a computerized random-sequence-generation program run with a permuted block design (block size of 4). Patients were matched based on BMI and age. The randomization list was given by a person not involved in the project. Supplements were packaged in equal containers and numbered with specific codes in order to disguise treatment allocation from research workers. All of the study staff and participants were blinded to treatment allocations and codes were given to the investigators after the statistical analyses.

### Assessment of outcomes

Fasting blood sugar (FBS), insulin resistance, insulin, TC, low-density lipoprotein (LDL-C), TG, high-density lipoprotein (HDL-C), tumor necrosis factor alpha (TNF-α) and Sirtuin1 (SIRT1) were recognized as the primary outcome. Body composition, waist circumference (WC), weight and hip circumference (HC) were considered as the secondary outcome.

### Anthropometric indices

The following factors were assessed at the outset and the end of the intervention. A stadiometer with a 0.1 cm accuracy was used to measure the height, a Seca digital scale with a 0.1 kg accuracy was used to measure weight (without shoes and lightweight clothing). A non-stretch tape with 0.5 cm accuracy was used to measure the hip circumference (HC) across largest part of the hips and the waist circumference (WC) at the higher iliac crest. Tanita MC-780 S MA, a bioimpedance analyzer, assessed the body composition of the patients (Amsterdam, the Netherlands).

### Blood sampling and biochemical measurements

Before and after the trial, 10 ml of venous blood was collected from each participant following 10–12 h of fasting. Before freezing the serum samples in a −80°C refrigerator, lipid profile and FBS were analyzed at the Jahad Medical Diagnostic and Pathology Laboratory. This laboratory used the kits of Pars-Azmoon Co., Tehran, Iran. William Fried Ewald’s formula was used to determine LDL-C level. Insulin resistance index was calculated using insulin and FBS data (HOMA-IR = (fasting glucose × fasting insulin) /405)). Serum insulin level was measured by commercial kits (Monobind, Lake Forest, CA, United States). Serum levels of Sirtuin1 and TNF-α were measured based on ELISA method with kits of Zelbio Co, Germany and LDN Co, Germany, respectively.

### Sample size

To determine the sample size, primary data including mean and standard deviation of HDL-C was obtained from Yoshida et al. study. The sample size with 95% confidence interval and power 85% was calculated using PASS software. The sample size was considered to be 22 people in each group, including a 20% attrition rate.

### Statistical analysis

All statistical data was analyzed using SPSS software version 20 (SPSS Inc., Chicago, Ill., United States). The Kolmogorov–Smirnov test was used to assess the normality of data distribution. Quantitative were reported as mean ± SD and median (Min, Max). Rank signed Wilcoxon test and a Paired t-test were utilized to analyze variable changes within-group. U Mann Whitney test and independent sample t-test were conducted to analyze variable changes between group. Analysis of covariance (ANCOVA) and quantile regression adjusted for confounding factors such as age, baseline values, and mean changes in weight was applied to determine the absolute effects of the therapy. A significant difference was indicated by *p* < 0.05.

## Results

### Characteristics of participants

**Figure 1:**
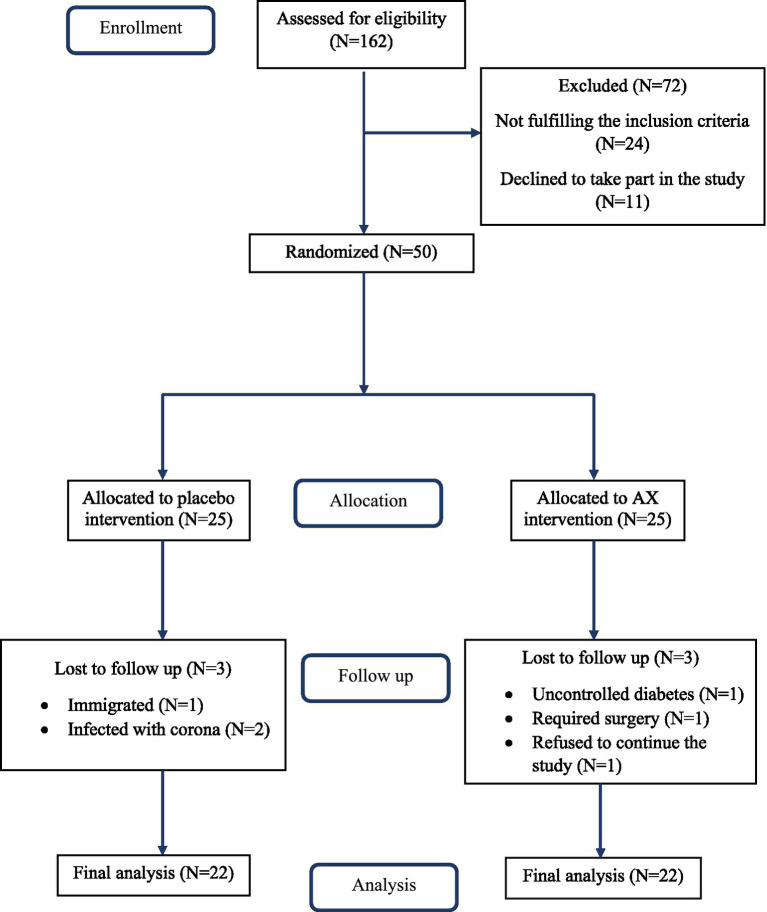
Flow diagram of study.

The average of age, weight, and BMI of all subjects were 58.22 ± 5.48 years, 86.12 ± 11.75 kg, and 30.10 ± 3.17 Kg/m^2^, respectively. Three participants were excluded from the placebo group, of whom one patient immigrated, and two patients were infected with COVID-19. Additionally, three individuals of the AX group were excluded due to uncontrolled diabetes, required surgery, or refused to continue the study. The intervention was completed by 22 individuals in each treatment group (Figure 1). Participants who took AX or placebo supplements did not report any side effects.

Distribution of age, BMI, gender, degree of physical activity and consumed drugs were not significantly different between the AX and placebo groups at the beginning of the study (Table 1).

**Table 1:** Data are described as means ± SD or number (%).

Baseline variables	Control group (*N* = 22)	AX group (*N* = 22)	value of *p*
Age	58.36 ± 5.85	58.09 ± 5.21	0.871^a^
*Gender*			
Men	17 (77.3)	19 (86.4)	0.698^b^
Women	5 (22.7)	3 (13.6)	
Weight	86.46 ± 12.52	85.78 ± 11.21	0.849^a^
BMI	30.45 ± 3.27	29.74 ± 3.11	0.465^a^
*Physical activity*			
Sedantary	4 (9.1)	8 (18.2)	0.77^b^
Moderate	14 (31.8)	14 (31.8)	
High	4 (9.1)	0 (0)	
*Drugs*			
Angiotensin-converting enzyme inhibitors	4 (30.8)	9 (69.2)	0.326^b^
Statins	22 (52.4)	20 (47.6)	
Angiotensin receptor blockers	13 (59.1)	9 (40.9)	
Beta-blockers	15 (48.4)	16 (51.6)	

a Independent sample *t*-test.

b Fisher’s exact test.

### Anthropometric measures

The comparison of anthropometric parameters of two groups was presented in Table 2. Baseline values of BMI, HC, WC and body composition were not significantly different between AX and placebo groups.

**Table 2:** Anthropometric parameters.

Variable		Placebo (*n* = 22)	Astaxanthin (*n* = 22)	MD, *p*
BMI (kg/m^2^)	before	30.45 ± 3.27	29.74 ± 3.11	−0.71, 0.465^*^
	after	29.99 ± 3.08	29.37 ± 3.15	−0.61, 0.515^*^
	MD, *p*^†^	−0.46,**0.005**	−0.03,**0.01**	0.95^●^
Body weight (kg)	before	86.46 ± 12.52	85.78 ± 11.21	−0.68, 0.849^*^
	after	85.17 ± 12.26	84.74 ± 11.04	−0.43, 0.903^*^
	MD, *p*^†^	−1.29,**0.003**	−1.04,**0.01**	0.68^●^
WC (cm)	before	107.45 ± 7.48	105.4 ± 7.08	−2.04, 0.357^*^
	after	106.97 ± 7.23	10,408 ± 6.94	−2.29, 0.289^*^
	*p*^†^	**0.04**	**0.04**	0.25^●^
HC (cm)	before	108.5 (101, 120)	108 (99,118)	0.638^§^
	after	108.5 (100,119)	108 (99,117)	0.646^§^
	*p* ^¥^	0.03	0.03	0.66^ᶿ^

BMI, Body mass index, WC, Waist circumference; HC, Hip circumference; Values are reported as mean ± standard deviation or median (minimum, maximum).

* Independent samples *t*-test.

† Paired samples *t*-test.

§ Mann–Whitney *U* test.^¥^Wilcoxon test.

● Adjusted for baseline values, age, and weight changes using the analysis of covariance (ANCOVA) test.

ᶿ Adjusted for baseline values, age, and weight changes using Quantile regression.

There was a significant reduction in WC and HC in both groups (*p* < 0.05) after 8 weeks. However, the changes between the two groups were not significant. In AX supplemented group and control group significant reduction of BMI (−1.24, −1.44%, respectively) and weight (−1.18, −1.46%, respectively) were observed, although between-group analyses did not show statistically difference.

### Body composition measures

The FFM (Fat-free mass) in the control group was reduced slightly more than AX group (−0.19% vs. –0.41%), with no significant within and between-group difference. Despite the significant reduction in visceral fat in the control group compared to the AX group at the end of the intervention, the between-group difference was not statistically significant. (*p* = 0.70). Fat mass and body fat percentage declined in both AX and placebo groups after 8 weeks. Although the results of the ANCOVA test did not show significant changes (*p* = 0.53, *p* = 0.41, respectively; Table 3).

**Table 3:** Body composition.

Variable		Placebo (*n* = 22)	Astaxanthin (*n* = 22)	MD, *p*
Body fat (%)	before	27.27 ± 7.35	25.57 ± 6.61	−1.70, 0.425^*^
	after	26.47 ± 7.52	25.01 ± 6.64	−1.44, 0.503^*^
	MD, *p*^†^	−0.80,**0.002**	−0.54,**0.01**	0.41^●^
Body fat (kg)	before	23.68 ± 7.30	22.07 ± 49.6	−1.60, 0.444^*^
	after	22.62 ± 7.15	21.29 ± 50.6	−1.32, 0.523^*^
	MD, *p*^†^	−1.05,**0.001**	−1.04,**0.01**	0.53^●^
FFM (kg)	before	78.62 ± 10/10	66.63 ± 96.8	0.88, 0,761*
	after	55.62 ± 29/10	46.63 ± 51.8	0.90, 0,753*
	*p*^†^	−0.23, 0.28	−0.20, 0.34	0.73^●^
Body water (%)	before	51.75 ± 5.29	52.83 ± 4.78	1.08, 0.480^*^
	after	52.38 ± 5.46	53.34 ± 4.70	0.95, 0.536^*^
	MD, *p*^†^	0.63,**0.004**	0.51,**0.01**	0.72^●^
Visceral fat	before	12.68 ± 3.06	12.63 ± 3.83	−0.45, 0.966^*^
	after	12.45 ± 2.98	12.36 ± 3.68	−0.45, 0.964^*^
	MD, *p*^†^	−0.27,**0.01**	−0.27, 0.08	0.70^●^

FFM, fat free mass; Values are reported as mean ± standard deviation.

* Independent samples *t*-test.

† Paired samples *t*-test.

● Adjusted for baseline values, age, and weight changes using the analysis of covariance (ANCOVA) test.

### Metabolic parameters

The baseline values of lipid profile and glycemic indices were not statistically different between two groups (Table 4). The reduction of TC in the AX group was significantly in contrast to the control group (−8.57% vs. –6.12%). Also, there was a significant decline in serum levels of LDL-C at the end of the intervention following AX supplementation unlike the control group (−13.59% vs. –7.49%). Although, the between-group difference of TC and LDL-C were not statistically significant. The results revealed that there was no significant difference in TG and HDL-C within and between groups. A 0.32% reduction in FBS in the AX group was observed compared with a 3.92% decrease in the control group; however, between group analysis did not show significant difference (*p* = 0.70). Independent t-test indicated no significant changes in the levels of insulin (*p* = 0.35) as well as HOMA-IR (*p* = 0.32).

**Table 4:** lipid profile and glycemic indices.

Variable		Placebo (*n* = 22)	Astaxanthin (*n* = 22)	MD, *p*
TC (mg/dl)	before	140.36 ± 32.82	155.86 ± 34.61	15.50, 0.135^*^
	after	129.81 ± 29.84	140.90 ± 37.12	11.09, 0.281^*^
	MD, *p*^†^	−10.54, 0.06	−14.95,**0.04**	0.88^●^
TG (mg/dl)	before	160.72 ± 72.89	146.31 ± 38.84	−14.40, 0.418^*^
	after	149.50 ± 55.33	145.90 ± 55.32	−3.59, 0.831^*^
	MD, *p*^†^	−11.22, 0.26	−0.40, 0.96	0.61^●^
HDL-C (mg/dl)	before	1.580 ± 0.10	1.618 ± 0.08	0.37, 0.197^*^
	after	1.581 ± 0.10	1.614 ± 0.08	0.32, 0.267^*^
	*p* ^ᵽ^	−0.0009, 0.93	−0.003, 0.80	0.84^●^
LDL-C (mg/dl)	before	69.03 ± 26.32	87.37 ± 30.30	15.33, 0.080^*^
	after	60.69 ± 2,380	69.72 ± 30.47	9.03, 0.279^*^
	MD, *p*^†^	−8.34, 0.06	−14.64,**0.02**	0.96^●^
FBS (mg/dl)	before	99.00 (89, 130)	97.00 (81, 130)	0.086^§^
	after	96.50 (71, 162)	97.00 (74, 130)	0.707^§^
	*p* ^¥^	0.053	0.592	0.91^ᶿ^
Insulin (μU / ml)	before	8.34 ± 3.98	7.47 ± 5.59	−0.86, 0.559^*^
	after	7.58 ± 4.01	6.20 ± 4.29	−1.16, 0.352^*^
	*p*^†^	−0.75, 0.06	−1.26, 0.07	0.62^●^
HOMA-IR	before	2.26 ± 1.23	1.92 ± 1.63	−0.34, 0.439^*^
	after	1.95 ± 1.15	1.58 ± 1.17	−0.35, 0.326^*^
	*p*^†^	−0.31, 0.07	−0.32, 0.13	0.31^●^

TC, Total Cholesterol; TG, Triglyceride; HDL-C, High-density lipoprotein-cholesterol; LDL-C, Low-density lipoprotein-cholesterol; FBS, Fasting blood sugar, Values are reported as mean ± standard deviation or median (minimum, maximum).

* Independent samples *t*-test.

† Paired samples *t*-test.

^§^Mann-Whitney U test. ^¥^Wilcoxon test.

● Adjusted for baseline values, age, and weight changes using the analysis of covariance (ANCOVA) test.

ᵽ *p* value is presented after logarithmic transformation.

^ᶿ^Adjusted for baseline values, age, and weight changes using Quantile regression.

### Sirtuin1 and TNF-α levels

The baseline values of Sirtuin1 and TNF-α were not statistically different between two groups (Table 5). There was a much more reduction in the levels of Sirtuin1 in the placebo group compared to AX group (−5.40% vs. −0.26%, *p* = 0.53). TNF-α levels did not change significantly in both groups. Based on between group analysis the levels of Sirtuin1 (*p* = 0.58) and TNF-α (*p* = 0.534) remained unaffected after 2 weeks.

**Table 5:** Sirtuin1 and TNF-α levels.

Variable		Placebo (*n* = 22)	Astaxanthin (*n* = 22)	MD, *p*
SIRT-1 (ng/ml)	before	6.37 (4.84, 9.62)	6.34 (5.12, 9.23)	0.733^§^
	after	5.90 (4.96, 9.84)	5.87 (4.57, 13.62)	0.534^§^
	*p* ^¥^	0.638	0.495	0.15^ᶿ^
TNF-α (pg/ml)	before	0.85 ± 0.27	0.88 ± 0.27	0.02, 0.763^*^
	after	0.85 ± 0.24	0.90 ± 0.26	0.04, 0.607^*^
	*p* ^ᵽ^	0.001, 0.986	0.02, 0.715	0.56^●^

SIRT-1, Sirtuin1; TNF-α, Tumor necrosis factor α, Values are reported as mean ± standard deviation or median (minimum, maximum).

* Independent samples *t*-test.

^§^Mann-Whitney U test. ^¥^Wilcoxon test.

● Adjusted for baseline values, age, and weight changes using the analysis of covariance (ANCOVA) test.

ᵽ *p* value is presented after logarithmic transformation.

^ᶿ^Adjusted for baseline values, age, and weight changes using Quantile regression.

## Discussion

*In vivo* studies have demonstrated that AX intake has cardiovascular protective effects, although the results of human studies regarding the beneficial effects of AX on cardiovascular risk factors are contradictory and limited. To the best of our knowledge, this study was the first randomized, double-blind, placebo controlled, clinical trial investigating the impacts of AX supplementation on metabolic parameters in CAD patients. The findings revealed that AX had no effect on the lipid profile compared to the placebo group, but it should be noted that LDL-C and TC levels decreased significantly in the AX group. However, it had no significant improvements on body composition and other metabolic and anthropometric parameters.

The results of a recent meta-analysis conducted in 2020 are in line with our findings and demonstrate that body weight and BMI changes were not statistically significant (23). Supplementation of 12-week AX (6 or 12 mg/d) in overweight middle-aged and senior subjects did not affect body weight and BMI (24). Choi et al. reported that 20 mg AX administration in overweight subjects for 12 weeks showed the same results (25). According to the food intake data, the energy intake of the patients in both groups decreased, as a result, BMI and body weight decreased in both groups. Despite of adjusting confounding factors, no substantial between-group changes were found. WC and HC are parameters that were only analyzed in this study, even though the differences between the AX-receiving group and the placebo group are not statistically significant.

Alike our results, Roustae Rad et al. investigated that lipid profile did not change significantly through consuming 10 mg AX after 12 weeks in T2DM subjects receiving metformin (26). Also, administration of 12 mg AX for 12 weeks in healthy volunteers including subjects with prediabetes exhibit the same outcomes (19). In contrast, in another clinical trial on T2DM patients revealed that 8 weeks AX administration at the dosages of 6 and 12 mg led to LDL-C reduction and only 12 mg AX cause TG and TC decrease (17). Another study on non-obese subjects with mild hyperlipidemia reported that 12 and 18 mg AX significantly diminish TG levels after 8 weeks (18). Two recent meta-analysis investigated that AX consumption was not associated with TC, LDL-C, TG reduction. However, only Xia reported an overall increase in HDL-C (23, 27). Tominaga et al. and Saito et al. showed that AX had no impact on lipid profile in healthy individuals regardless of the duration of supplementation and the AX dosage (28, 29).

It is well known that the major causative risk factors for CAD and a definite contributor to accelerated atherosclerosis is dyslipidemia (30, 31). Statins are used as the first line of treatment in both primary and secondary prevention to lower cholesterol and enhance lipid composition (32, 33). Therefore, AX might not have affected TC, TG, and LDL-C levels since the majority of the patients were taking statins and lipid profile had improved before the intervention.

However, the TC and LDL-C levels were significantly decreased only in the intra-group analysis of AX group. The capacity of AX to upregulate SREBP-2 and therefore boost the gene expression of LDLR and HMGR may have contributed to the ability of AX to reduce cholesterol levels (34). SIRT1 involve in regulating the homeostasis of the body’s total cholesterol as a positive regulator of the liver X receptor (LXR) (35). It has been demonstrated that SIRT1 also regulates PPARα expression, which helps to control lipid metabolism (36, 37).

The current study also declared that AX intake did not significantly improve serum levels of insulin, FBS, and HOMA-IR index. A recent meta-analysis revealed that FBS did not decrease after AX supplementation compare with placebo group. According to yang et al. study, the plasma levels of glucose in apoE knockout (apoE^−^/^−^) mice, a mouse model for atherosclerosis, which were fed a high-fat/high-cholesterol diet supplemented with AX-rich Hematococcus pluvialis extract for 4 weeks did not improved significantly compared with control group (34). Alike our result, Saito and Tominaga et al. demonstrated that 12 mg, 6 and 12 mg AX did not affect FBS levels in healthy volunteers, respectively (28, 29). Yoshida et al. also reported the similar result which FBS did not change significantly in subjects with mild hyperlipidemia (18). However, the blood glucose levels have dropped significantly or even slightly in trials on diabetic subjects (16, 17).

SIRT1 play an important role in controlling cellular physiological processes (38). It has been identified as a novel homoeostasis regulator in the human cardiovascular system through protecting against aging and endothelial inflammation, inducing resistance against oxidative stress and hypertrophic, inhibiting apoptosis of cardiomyocytes, and modulating cardiac energy metabolism (39, 40). Furthermore, transcription factor SIRT1 plays an important role in energy and lipid metabolism, inflammation and insulin sensitivity (41). Nishida et al. reported that AX treatment induced upregulation of the gene expression of the mitochondrial SRIT1 and mitochondrial biogenesis in mice (42). In our investigation no significant change in serum level of SIRT1 following AX supplementation were observed even after adjusting for confounders. Non-significant alterations in lipid profile and glycemic indices may be associated with negligible changes in SIRT1. The impact of AX on SIRT1 levels has not been studied in any RCT. Furthermore, drawing a definite conclusion on the effects of AX on SIRT1 is not feasible due to the insufficient number of studies in this field.

TNF-α is a pro-inflammatory cytokine found in atherosclerotic lesions and can have a direct effect on vascular endothelial cells and induce the expression of adhesive molecules in leukocytes and other inflammatory cells (43). In our study the changes of TNF-α levels were not significant and in line with our study. Park et al. demonstrated that AX administration at two doses of 2 and 8 mg for 8 weeks did not affect TNF-α in healthy women (44). Although, 6 and 12 mg AX consumption for 8 weeks in diabetic patients could alleviate diabetes-associated systemic inflammatory stress by reducing TNF-α circulation (17). Considering that more than 80% of the participants in our study had normal range of TNF-α at the baseline, a higher dose should probably be used to have a greater impact on TNF-α factor. Alternatively, the result of non-significant change in TNF-α levels in our study is in line with the hypothesis of the recent meta-analysis about the effect of carotenoids supplementation on inflammation, which suggests that higher baseline levels of pro-inflammatory cytokines indicate a potential for a greater response to carotenoid supplementation such as AX (45).

SIRT1 directly can deacetylate the p65 subunit of the NF-κB complex to suppress NF-κB activation. Furthermore, SIRT1 promotes oxidative energy generation by activating of AMP-activated protein kinase (AMPK), Peroxisome proliferator-activated receptor α (PPARα) and Peroxisome proliferator-activated receptor gamma coactivator 1-alpha. These factors concurrently inhibit NF-κB signaling and led to decline inflammation (46). If this pathway is blocked, TNF-α production can also decrease.

The small sample size may be the reason for the lack of noticeable changes in the parameters of present study. The follow-up duration in our study was short and extending the duration of the intervention might show potential benefits. Moreover, we were not able to measure the concentration of AX in the plasma in order to evaluate its bioavailability. Future trials may be able to better understand the impacts of AX by assessing certain genes involved in the regulation of metabolic variables.

The trial was completely conducted using a double-blind, random allocation approach. Patients who had recovered from COVID-19 even before the intervention were excluded from the study due to prevent disturbances in the measured factors, especially the inflammatory index.

## Conclusion

The data indicate that the levels of LDL-C and TC have decreased after using AX supplements for 8 weeks, while other metabolic variables have not significantly changed. However, more clinical trials with various AX supplementation doses and durations are required to obtain a definitive conclusion.

## Data availability statement

The raw data supporting the conclusions of this article will be made available by the authors, without undue reservation.

## Ethics statement

The studies involving human participants were reviewed and approved by Tabriz University of medical science. The patients/participants provided their written informed consent to participate in this study.

## Author contributions

MH, MA, and MC designed the study. MH, MC, and BS coordinated the collection of the samples and data collection from study participants. MH carried out the statistical analysis and wrote the first draft of the article. MH, MC, MA, BS, and SK participated in revising it critically for important intellectual content. All authors contributed to the article and approved the submitted version.

## Funding

Tabriz University of Medical Sciences supported this study (grant number 65868).

## Conflict of interest

The authors declare that the research was conducted in the absence of any commercial or financial relationships that could be construed as a potential conflict of interest.

## Publisher’s note

All claims expressed in this article are solely those of the authors and do not necessarily represent those of their affiliated organizations, or those of the publisher, the editors and the reviewers. Any product that may be evaluated in this article, or claim that may be made by its manufacturer, is not guaranteed or endorsed by the publisher.
